# Spinal Extension by Suspension*Read before the Buffalo Medical Club

**Published:** 1882-08

**Authors:** Bernard Bartow


### BUFFALO

### Medical and Surgical Journal.

VOL. XXII. AUGUST, 1882. No. 1.

### Original Communications.

### SPINAL EXTENSION BY SUSPENSION.*

BY BERNARD BARTOW, M. D.

The idea advanced in connection with the plaster-of-Paris jacket for the treatment of vertebral caries, viz., to correct spinal mal-position by suspension of the patient, previous to the fixation of the spinal column with the plaster bandage, has been before the profession long enough to determine upon what merit the practice stands. Regarding the correctness of the principle involved in the plaster-of-Paris jacket for effecting spinal fixation, and immobility of the diseased vertebræ, there is no dissent; of the value of forced spinal extension as a preliminary measure, there has been a free expression of opinion as to its correctness, employed in the manner recommended by its author. Notwithstanding this, it is a matter of surprise at this time, that there should be medical men imbued with sufficient faith in, or possessing such false conceptions of, a particular plan of treatment, as to believe that an angular deformity of a part of the spinal column, is capable of being restored without injury to the patient; who can see nothing irrational in a plan of treat-

*Read before the Buffalo Medical Club ment, that by forcible spinal stretching attempts, apparently, to diminish the curve in a part of the column, the result of a destructive disease of some of its bones. That there are medical men who hold such views, and practice in accordance with them, has been frequently verified in my experience; such conclusions have been formed, in part, by their expression of opinion, and the results of their practice, and in part, by the statements of patients regarding the manner of their treatment previous to coming under my care. I have seen several bad results following suspension in spinal caries; in each instance there was increased inflammatory action at the seat of disease, and, notwithstanding the application of the plaster jacket, increase of the angular deformity. The statements that are commonly made to me by patients who have been treated by this method, are, that the suspension was carried to the point fully to lift them off their feet, that it was painful, that fainting followed in some instances, and, that after several weeks they were worse than before the treatment was begun. Making due allowance for changes incident to the natural course of the disease, I am forced to believe that there is a large proportion of truth in such statements; that an amount of injury has been inflicted upon persons with spinal caries, directly due to the forcible extension so indiscriminately practiced in every case that has the semblance of spinal disease, previous to applying the plaster of Paris jacket.

The cause for such wide misapplication of what, properly used, is a valuable adjunct to the mechanical treatment of spinal disease, is due, it seems to me, to two facts: 1st, indefinite ideas on the part of many practitioners of the nature and effect of vertebral ostitis, and an inability to recognize the different stages of the disease; 2nd, to the imperfect manner in which the method of treatment that includes suspension, has been promulgated. Of the former, it is to be deplored that there are many physicians who fail in the recognition of spinal disease in its early stage. With little appreciation of the loss that follows to the patient from this oversight, the disease is permitted to progress until permanent deformity, more or less extensive, has shown itself; then the diagnosis is made, and often it is considered to have been an early diagnosis of the disease, and as such, offering advantages in the matter of preventing deformity. With such views of a case, is it surprising that the same practitioner, having faith in himself to follow to the letter the rules of treatment, should hang up his patient by the head and axillæ, stretch out the spine until all trace of deformity has been removed, encase him in plaster-of-Paris bandages, and then congratulate himself on the good result he is taught to expect? In regard to the 2nd, Dr. Sayre (who has given the impetus to this mode of treatment) in his book, "Spinal Disease and Spinal Curvature," (page 8), says: "The patient should not be permitted to assume the upright position before he has been fitted with some artificial support capable of removing all pressure from the bodies of the diseased vertebræ. This object may be accomplished by *straightening** the spinal column in such a manner that the weight of the body is borne by the transverse processes." Speaking of lifting apart the bodies of the diseased vertebræ by mechanical appliances (page 8), he says: "This can be done only by an accurately fitting apparatus applied to the body itself when *extended*." * Describing the method of measuring the abnormal curves before and during suspension, with a view to showing the improvement in spinal position, due to suspension, (page 15), he says: "Thus we have a means by which comparison can be made, and are enabled to determine exactly what changes have taken place in the *curve*." * In making spinal extension by suspension with the head-and-chin collar and axillary supports, he recommends that the patient should be "gradually drawn up until his feet swing just clear of the floor."

Although Dr. Sayre appends a word of caution upon the impossibility of attempting to straighten curved spines due to caries, in which repairative processes are active, or have consolidated the bones, he nevertheless, by his illustration of the subject,

*Italics mine.

shows advanced caries of the spine subjected to the suspension treatment, previous to application of the plaster jacket. Further, there is nothing mentioned by him, to indicate to those following his instructions, that there is a period of the disease in which there is *no* angular projection of a vertebræ; that there is *no* period after posterior projection of a spinous process has shown itself, however small the deformity may be, when repairative efforts are not active; that suspension, in the manner advised, any time after posterior projection has taken place, might seriously disturb processes that are essential to the arrest of the disease. No reference is made to secondary or compensating curves that are induced in normal portions of the column, their effect to increase the deformity, or how, by their removal, the permanent deformity is *apparently* lessened.

The inferences to be drawn from Dr. Sayre's statements, by one who was relying upon his teaching for a knowledge of the subject, would be: That the improvement of spinal position induced by suspension, was due to a change in the direction of the vertebræ adjacent to and including the seat of disease; that a lifting apart, or separation, of the surfaces of the vertebral bodies most intimately involved in the carious process was to be effected by suspension—producing a gap on the anterior aspect of the column. A somewhat similar idea is expressed by Barwell.* Referring to Dr. Sayre's method, he says: "The idea is simple enough, merely to suspend the patient for a few minutes by the arm-pits and head, so that the weight of the lower limbs and buttocks shall *straighten out*† to some considerable degree the bend in the spine."

It is no exaggeration to say that the views of the majority of practitioners, as derived from these teachings, are in accord with the inferences just mentioned. As opposed to such teaching, it seems almost superfluous to direct attention to a few cardinal facts in the pathology of the disease; That absorption of part or whole

*Curvatures of the Spine, Lond., 1877.

† Italics mine.

of a vertebral body necessitates proportionate posterior projection of its spinous process; That there are present from the early stage of the disease until consolidation of the carious bones is effected, conservative processes of an inflammatory nature, affecting the periosteum, ligaments, etc. contiguous to the seat of disease, whose tendency is to immobilize that part of the column; in the natural course of the disease those efforts are usually inadequate to its arrest, but deformity being present, nothing should be done to disturb those attempts of nature to oppose the progress of the disease; That an abnormal angle assumed by the diseased vertebræ must be allowed to remain,—apposition of the surfaces of the carious vertebral bodies being essential to union and firm anchylosis of the parts; That improvement of spinal mal-position due to caries can, properly, take place by correcting compensating curves only. Such facts seem wholly to have been lost sight of, in the rush for brilliant results, in the treatment of this affection, instead of accepting deformity, when present, as a necessary evil, and turning attention to its prevention rather than its cure.

The rule, given by Dr. Sayre, for suspending a patient with spinal caries, is a dangerous one for general application, even with such protective modifications as have been added to it; for, in the present state of the matter, it is probable that in eighty per cent. of the cases so treated, there would be angular deformity present, in varying degrees, at the commencement of treatment.

There is but one stage of vertebral ostitis, in which suspension, as he recommends it, would be admissible, viz., before absorption begins in the vertebral body, or cartilage; previous to the occurrence of absorption there is lordosis, induced by reflex irritation, that can be wholly overcome by suspension, without danger of aggravating the inflammatory action that, in a little more advanced stage of the disease, constitutes a strong objection to its use. Slight abnormal projection of a spinous process, ensuing upon lordosis, points directly to commencing destruction of a vertebral body, and is an indication for extreme care in the use of a force that may violently disturb the parts. With patients who have pronounced angular curvature, of whatever degree, it is *never* necessary to suspend them so that the feet swing clear of the floor; it is rarely necessary even to raise them off their heels to correct compensating curves. As a rule it will be found that by a forced muscular effort to erect his spine, the patient can improve its position quite as effectually as by employing suspension.

Previous to the application of a fixation apparatus, such improvement of spinal position as a case permits, can be accomplished by directing the patient to erect his spine, giving him at the same time slight axillary support.

By these means, the plaster-of-Paris bandage can be applied with all the advantages the method admits, while the danger of possible harm to the patient is wholly removed. In children too young to assist themselves, it is necessary for the surgeon to determine the degree of suspension to be used. In a case of slight angular projection of a spinous process, there will be a temptation to suspend the patient to the extent to cause *all* deformity to disappear; this, from the yielding character of the tissues, can be easily accomplished, but the evil of such a course will be seen in the certain increase of the inflammatory action that will shortly ensue. It is a nice point, therefore, in such an instance, to decide upon the degrees of force with which suspension should be exerted. The only arbitrary rule that can be stated safely, is:—Avoid disturbing an *angle* in the vertebral axis. This may be formed by the projection of but one spinous process, but nevertheless, it is a *sure* indication of loss of tissue in one, or between two vertebral bodies. The continuity of the anterior segment of the column can be secured only by permitting the fusion of two vertebral bodies, in one or both of which the disease may be located. A method of treatment that contemplates anything but this, is devoid of safety and reason. It is earnestly to be desired that more attention be given to the early recognition of this disease, that event alone holding out a reasonable expectation of success in the prevention of deformity. As a rule, these cases, in their incipiency, come under the notice of the family physician, and it is with him that much of the responsibility rests as to the future comfort or unhappiness of the patient.

